# Supplementary material for: Family as a health promotion setting: A scoping review of conceptual models of the health-promoting family
Source: PLoS One. 2021 Apr 12;16(4):e0249707. doi: 10.1371/journal.pone.0249707 (PMC8041208; doi:10.1371/journal.pone.0249707)
Supplement: S1 Table — (DOCX) [file pone.0249707.s001.docx]

**S1 Table. HPF review evidence table.**

**List of included documents**

| Doc # | Reference |
| --- | --- |
|  | Aldossari, N. Agents of their health? Mothers as agents of children’s oral health in the kingdom of Saudi Arabia. PhD Dissertation, University of Sheffield. 2016. Available from: <http://etheses.whiterose.ac.uk/15717/1/Saudia%20version%20(1).pdf>. |
|  | Annim SK, Awusabo-Asare K, Amo-Adjei J. Household nucleation, dependency and child health outcomes in Ghana. Journal of Biosocial Science. 2015;47(5): 565-592. doi: 10.1017/s0021932014000340. |
|  | Armstrong MI, Birnie-Lefcovitch S, Ungar MT. Pathways between social support, family well- being, quality of parenting, and child resilience: what we know. Journal of Child and Family Studies. 2005;14(2): 269-281. |
|  | Ashiabi GS, O'Neal KK. Children's health status: examining the associations among income poverty, material hardship, and parental factors. PLoS One. 2007;2(9): e940. |
|  | Ayala GX, Carnethon M, Arredondo E, Delamater AM, Perreira K, Van Horn L, et al. Theoretical foundations of the study of Latino (SOL) youth: implications for obesity and cardiometabolic risk. Annals of Epidemiology. 2014;24(1) :36-43. |
|  | Baiocchi-Wagner EA, Talley AE. The role of family communication in individual health attitudes and behaviors concerning diet and physical activity. Health Communication. 2013;28(2): 193-205. |
|  | Ball J, Moselle K, Pedersen S. Father's involvement as a determinant of child health. In: Public Health Agency of Canada, Population Health Fund Project: Father Involvement for Healthy Child Outcomes: Partners Supporting Knowledge Development and Transfer*,* March 1, 2007. Available from: <http://www.ecdip.org/docs/pdf/PH%20FI%20Final%20Full%20Report.pdf>. |
|  | Bates CR, Bohnert AM, Buscemi J, Vandell DL, Lee KTH, Bryant FB. Family entropy: understanding the organization of the family home environment and impact on child health behaviors and weight. TBM. 2019;9: 413-421. doi: 10.1093/tbm/ibz042. |
|  | Batorwicz B, King G, Mishra L, Missiuna C. An integrated model of social environment and social context for pediatric rehabilitation. Disability and Rehabilitation. 2016;38(12): 1204-1215. doi: 10.3109/09638288.2015.1076070. |
|  | Berkel C, Maurico AM, Sandler IN, Wolchik SA, Gallo CG, Brown CH. The cascading effects of multiple dimensions of implementation on program outcomes: a test of a theoretical model. Prevention Science. 2018;19: 782-794. |
|  | Bertrand L. Characterizing the school food environment in Dominica for health promotion. PhD Dissertation, University of Saskatchewan. 2019. Available from: <https://harvest.usask.ca/handle/10388/12119>. |
|  | Bomar PJ. Introduction to family health nursing and promoting family health. In Bomar PJ. Promoting health in families: applying family research and theory to nursing practice. 3rd ed. Philadelphia, Pennsylvania: Saunders, Elsevier; 2004. pp. 3-37. |
|  | Brown O, Fouche P, Coetzee M. Bouncing forward: families living with a type I diabetic child. South African Family Practice. 2010:52(6): 536-541. |
|  | Brug J, te Velde SJ, Chinapaw MJ, Bere E, De Bourdeaudhuij I, Moore H, et al. Evidence-based development of school-based and family-involved prevention of overweight across Europe: the ENERGY-project's design and conceptual framework. BMC Public Health. 2010;10(1): 276. |
|  | Butler AM. Social determinants of health and racial/ethnic disparities in Type 2 diabetes in youth. Curr Diab Rep. 2017;17: 60. doi: 10.1007/s11892-017-0885-0. |
|  | Cano MA, Castillo LG, Huang S, Romero AJ, Cordova D, Lizzi KM et al. Health risk behaviors and depressive symptoms among Hispanic adolescents: examining acculturation discrepancies and family functioning. Journal of Family Psychology. 2016;30(2): 254-265. doi: 10.1037/fam0000142. |
|  | Cheng ER. The impact of early childhood cognitive limitations on children and their families: evidence from the Early Childhood Longitudinal Study, Birth Cohort. University of Wisconsin: ProQuest LLC; 2013. |
|  | Chiu HJ. A test of the Bruhn and Parcel Model of health promotion. Journal of Nursing Research. 2005;13(3): 184-196. |
|  | Choy C, Isong IA. Assessing preschoolers’ beverage consumption using the Theory of Planned Behavior. Clinical Pediatrics. 2018;57(6): 711-721. doi: 10.1177/0009922817737076. |
|  | Christensen P. The health-promoting family: a conceptual framework for future research. Social Science & Medicine. 2004;59(2): 377-387. |
|  | Columna L, Dillon SR, Dolphin M, Streete DA, Hodge SR, Myers B, et al. Physical activity participation among families of children with visual impairments and blindness. Disability and Rehabilitation. 2019;41(3): 357-365. doi: 10.1080/09638288.2017.1390698. |
|  | Coviak CP. Child-parent reciprocal influences in exercise behavior. PhD Dissertation, University of Michigan. 1998. Available from: <http://gateway.proquest.com/openurl?url_ver=Z39.88-2004&rft_val_fmt=info:ofi/fmt:kev:mtx:dissertation&res_dat=xri:pqm&rft_dat=xri:pqdiss:9825192>. |
|  | Dai YT. The effects of family support, expectation of filial piety, and stress on health consequences of older adults with diabetes mellitus. PhD Dissertation, University of Washington. 1995. Available from: <http://hdl.handle.net/1773/7214>. |
|  | Davies PT, Sturge-Apple ML, Martin MJ. Family discord and child health: an emotional security formulation. In Families and child health. New York: Springer; 2013. pp. 45-74. |
|  | Davison KK, Jurkowski JM, Lawson HA. Reframing family-centred obesity prevention using the Family Ecological Model. Public Health Nutrition. 2013;16(10): 1861-1869. |
|  | De Coster S, Zito R. Maternal roles and adolescent depression: conditions and processes of influence. Sociological Perspectives. 2013:56(1): 1-23. |
|  | DeLorme AL, Gavenus ER, Charles RS, Benard GO, Mattah B, Bukusi E, et al. Nourishing networks: a social-ecological analysis of network intervention for improving household nutrition in Western Kenya. Social Science & Medicine. 2018;197: 95-103. doi: 10.1016/j.socscimed.2017.11.023. |
|  | Denham SA. Family health: a framework for nursing. Philadelphia: F.A. Davis Publishers; 2003. |
|  | dePaula JS, Ambrosano GMB, Mialhe FL. The impact of social determinants on schoolchildren’s oral health in Brazil. Braz Oral Res. 2015;29(1): 1-9. doi: 10.1590/1807-3107BOR-2015.vol29.0098. |
|  | Dion MR, Devaney B, McConnell S, Ford M, Hill H, Winston P. Helping unwed parents build strong and healthy marriages: a conceptual framework for interventions. Washington, DC: Mathematica Policy Research, Inc.; 2003. |
|  | dos Santos Costa F, Agostini BA, Schuch HS, Correa MB, Goettems ML, Demarco FF. Parent-child interaction and stimulation in early life can be related to caries in primary dentition? Hypotheses from a life-course approach. Medical Hypotheses. 2019;130. doi: 10.1016/j.mehy.2019.109291. |
|  | Drotar D, Bonner MS. Influences on adherence to pediatric asthma treatment: a review of correlates and predictors. Journal of Developmental & Behavioral Pediatrics. 2009;30(6): 574-582. |
|  | Dubowitz H, Newton RR, Litrownik AJ, Lewis T, Briggs EC, Thompson R, et al. Examination of a conceptual model of child neglect. Child Maltreatment. 2005;10(2): 173-189. |
|  | Duijster D, Loveren C, Dusseldorp E, Verrips GH. Modelling community, family, and individual determinants of childhood dental caries. European Journal of Oral Sciences. 2014;122(2): 125-133. |
|  | Dush CMK, Schmeer KK, Taylor M. Chaos as a social determinant of child health: reciprocal associations. Social Science & Medicine. 2013;95: 69-76. |
|  | East PL, Khoo ST. Longitudinal pathways linking family factors and sibling relationship qualities to adolescent substance use and sexual risk behaviors. Journal of Family Psychology. 2005;19(4): 571. |
|  | Ergler CR. The power of place in play: a Bourdieusian analysis of seasonal outdoor play practices in Auckland children’s geographies. PhD Dissertation, The University of Auckland. 2012. Available from: <https://researchspace.auckland.ac.nz/handle/2292/19737>. |
|  | Fairbrother H, Curtis P, Goyder E. Making health information meaningful: children’s health literacy practices. SSM-Population Health. 2016;2: 476-484. doi: 10.1016/j.ssmph.2016.06.005. |
|  | Farhood LF. Testing a model of family stress and coping based on war and non-war stressors, family resources and coping among Lebanese families. Archives of Psychiatric Nursing. 1999;13(4): 192-203. |
|  | Fisher-Owens SA, Gansky SA, Platt LJ, Weintraub JA, Soobader MJ, Bramlett MD, et al. Influences on children's oral health: a conceptual model. Pediatrics. 2007;120(3): e510-e520. |
|  | Friedemann ML. The framework of systemic organization: A conceptual approach to families and nursing. Thousand Oaks, California: Sage Publications Inc.; 1995. |
|  | Galbraith-Gyan KV, Lechuga J, Jenerette CM, Palmer MH, Moore AD, Hamilton JB. HPV vaccine acceptance among African-American mothers and their daughters: an inquiry grounded in culture. Ethnicity & Health. 2019:24(3): 323-340. doi: 10.1080/13557858.2017.1332758. |
|  | Golan M, Weizman A. Familial approach to the treatment of childhood obesity: conceptual model. Journal of Nutrition Education. 2001;33(2): 102-107. |
|  | Gold JI, Treadwell M, Weissman L, Vichinsky E. An expanded transactional stress and coping model for siblings of children with sickle cell disease: family functioning and sibling coping, self‐efficacy and perceived social support. Child: Care, Health and Development. 2008;34(4): 491-502. |
|  | Guagliano JM, Brown HE, Coombes E, Hughes C, Jones AP, Morton KL, et al. The development and feasibility of a randomised family-based physical activity promotion intervention: the Families Reporting Every Step to Health (FRESH) study. Pilot and Feasibility Studies. 2019a;5: 21. doi: [10.1186/s40814-019-0408-7](https://doi.org/10.1186/s40814-019-0408-7). |
|  | Guagliano JM, Brown HE, Coombes E, Haines ES, Hughes C, Jones AP, et al. Whole family-based physical activity promotion intervention: the families reporting every step to health pilot randomised controlled trial protocol. BMJ Open. 2019;9: e030902. doi: 10.1136/bmjopen-2019-030902. |
|  | Gunn HE, Eberhardt KR. Family dynamics in sleep health and hypertension. Current Hypertension Reports. 2019b;21: 39. doi: 10.1007/s11906-019-0944-9. |
|  | Hardt M, Jobe-Shields L, Williams JL. Emotional security theory: an application to sibling bereavement. Death Studies. 2019:43(10): 656-664. doi: 10.1080/07481187.2018.1511637. |
|  | Hauser-Cram P, Warfield ME, Shonkoff JP, Krauss MW, Sayer A, Upshur CC, et al. Children with disabilities: a longitudinal study of child development and parent well-being. Monographs of the Society for Research in Child Development. 2001: i-126. |
|  | Hendrick EC, Cohen AK, Deardoff J, Cance JD. Biological and sociocultural factors during the school years predicting women’s lifetime educational attainment. Journal of School Health. 2016;86(3): 215-224. doi: 10.1111/josh.12368. |
|  | Hendrie GA, Coveney J, Cox DN. Defining the complexity of childhood obesity and related behaviors within the family environment using structural equation modelling. Public Health Nutrition. 2012;15(1): 48-57. |
|  | Henton PA. A call to reexamine quality of life through relationship-based feeding. American Journal of Occupational Therapy. 2018;72(3): 7203347010. doi: 10.5014/ajot.2018.025650. |
|  | Holmes LG, Himle MB, Strassberg DS. Parental romantic expectations and parent-child sexuality communication in autism spectrum disorders. Autism: The International Journal of Research & Practice. 2016;20(6): 687-699. doi: 10.1177%2F1362361315602371. |
|  | Ickes SB, Heymsfield GA, Wright TW, Baguma C. “Generally the young mom suffers much:” socio-cultural influences of maternal capabilities and nutrition care in Uganda. Maternal & Child Nutrition. 2017;13: e12365. doi: 10.1111/mcn.12365. |
|  | Janin MMH, Ellis SJ, Lum A, Wakefield CE, Fardell JE. Parents’ perspectives on their child’s social experience in the context of childhood chronic illness: a qualitative study. Journal of Pediatric Nursing. 2018;42: e10-e18. doi: 10.1016/j.pedn.2018.06.010. |
|  | Jonsson L, Berg C, Larsson C, Korp P, Lindgren EC. Facilitators of physical activity: voices of adolescents in a disadvantaged community. International Journal of Environmental Research and Public Health. 2017;14: 839. doi: 10.3390/ijerph14080839. |
|  | Kalil AB. Caries experience of preschool children in selected sites in Johannesburg. PhD Dissertation, University of the Witwatersrand, South Africa. 2017. Available from: <http://wiredspace.wits.ac.za/handle/10539/24219>. |
|  | Kiefner-Burmeister AE, Hoffmann DA, Meers MR, Koball AM, Musher-Eizenman DR. Food consumption by young children: a function of parental feeding goals and practices. Appetite. 2014;74: 6-11. |
|  | Kim Seow W. Environmental, maternal, and child factors which contribute to early childhood caries: a unifying conceptual model. International Journal of Paediatric Dentistry. 2012;22(3): 157-168. |
|  | Kim-Godwin YS, Bomar PJ. Family health promotion. In: Kaakinen JR, Coehlo DP, Gedally-Duff V, Hanson SMH, editors. Family Health Care Nursing. 4th ed. Philadelphia: F.A.; 2010. pp. 209-213. |
|  | King G, Lawm M, King S, Rosenbaum P, Kertoy MK, Young NL. A conceptual model of the factors affecting the recreation and leisure participation of children with disabilities. Physical & Occupational Therapy in Pediatrics. 2003;23(1): 63-90. |
|  | Kitzman-Ulrich H, Wilson DK, George SMS, Lawman H, Segal M, Fairchild A. The integration of a family systems approach for understanding youth obesity, physical activity, and dietary programs. Clinical Child and Family Psychology Review. 2010;13(3): 231-253. |
|  | Koen V, van Eeden C, Rothmann S. A model for psychosocial well-being of families in a South African context. Journal of Psychology in Africa. 2013;23(1): 155-164. |
|  | Kumar A, Ram F. Influence of family structure on child health: evidence from India. Journal of Biosocial Science. 2013;45(5): 577-599. |
|  | Laing K, McWhirter J, Templeton L, Hannah-Russel C. M-PACT+: supporting families affected by parental substance misuse. Health Education. 2019;119(1): 63-82. |
|  | Lam Yuk Yin, W. The health literacy of Hong Kong Chinese parents with a healthy preschool child in seasonal influenza prevention and their health promotion strategies at the household level. PhD Dissertation, University of Technology Sydney. 2016. Available at: <https://opus.lib.uts.edu.au/handle/10453/90008>. |
|  | Larsen JK, Hermans RC, Sleddens EF, Engels RC, Fisher JO, Kremers SP. How parental dietary behavior and food parenting practices affect children's dietary behavior. Interacting sources of influence? Appetite. 2015;89: 246-257. doi: 10.1016/j.appet.2015.02.012. |
|  | Larsen KJ, Hermans RCJ, Sleddens EFC, Vink JM, Kremers SPJ, Ruiter ELM, et al. How to bridge the intention-behavior gap in food parenting: automatic constructs and underlying techniques. Appetite. 2018;123: 191-200. doi: 10.1016/j.appet.2017.12.016. |
|  | Lawton KE, Gerdes AC. Acculturation and Latino adolescent mental health: Integration of individual, environmental, and family influences. Clinical Child and Family Psychology Review. 2014;17(4): 385-398. |
|  | Lindsay AC, Greaney ML, Wallington SF, Mesa T, Salas CF. A review of early influences on physical activity and sedentary behaviors of preschool-aged children in high-income countries. Journal for Specialists in Pediatric Nursing. 2017;22: e12182. doi: 10.1111/jspn.12182. |
|  | Liu X. Parenting practices and the psychological adjustment of children in rural China. Gansu Survey of Children and Families Dissertations, University of Pennsylvania: Scholarly Commons; 2003. |
|  | Lopez-Rodriguez L, Navas M, Cuadrado I, Tatar M. Adjustment outcomes of Native and immigrant youth in Spain: a mediation model. The Spanish Journal of Psychology. 2018;21(e19): 1-10. doi: 10.1017/sjp.2018.19. |
|  | Majdandžić M, de Vente W, Feinberg ME, Aktar E, Bögels SM. Bidirectional associations between coparenting relations and family member anxiety: a review and conceptual model. Clinical Child Family Psychology Review. 2012;15: 28-42. |
|  | Mallette JK, O’Neal CW, Richardson EW, Mancini JA. When fathers are involved: examining relational and psychosocial health among military families. Family Process. 2020;X(X). doi: 10.1111/famp.12566. |
|  | Mammen J, Rhee H, Norton SA, Butz AM, Halterman JS, Arcoleo K. An integrated operational definition and conceptual model of asthma self-management in teens. Journal of Asthma. 2018;55(12): 1315-1327. doi: 10.1080/02770903.2017.1418888. |
|  | Markwalter DW, Murphy MA, Turnbull JM, Fanning JB. Framing the future: family preparedness for care transitions of critically ill children. American Psychological Association. 2019:37(3): 212-223. doi: 10.1037/fsh0000431.supp. |
|  | Meinzer MC, Hill RM, Pettit JW, Nichols-Lopez KA. Parental support partially accounts for the covariation between ADHD and depressive symptoms in college students. Journal of Psychopathology and Behavioral Assessment. 2015;37(2): 247-255. |
|  | Mindell JA, Williamson A.A. Benefits of a bedtime routine in young children: sleep, development, and beyond. Sleep Medicine Reviews. 2018;40: 93-108. doi: 10.1016/j.smrv.2017.10.007. |
|  | Molborn S, Lawrence E. Family, peer, and school influences on children’s developing health lifestyles. Journal of Health and Social Behavior. 2018;59(1): 133-150. doi: 10.1177%2F0022146517750637. |
|  | Moraes ES, Mendes-Castillo AMC. The experience of grandparents of children hospitalized in pediatric intensive care unit. Journal of School of Nursing. 2018;52(e03395): 1-8. doi: 10.1590/S1980-220X2017040003395. |
|  | Niermann CYN, Gerards SMPL, Kremers SPJ. Conceptualizing family influences on children’s energy balance-related behaviors: levels of interacting family environmental subsystems (The LIFES Framework). International Journal of Environmental Research and Public Health. 2018;15: 2714. doi: 10.3390/ijerph15122714. |
|  | Noonan-Gunning SE. Food-related obesity policy, parents and class: a critical policy analysis exploring disconnect. Unpublished Doctoral thesis, University of London. 2018. Available from: <https://openaccess.city.ac.uk/id/eprint/20096/>. |
|  | Oddo VM, Surkan PJ, Hurley KM, Lowery C, de Ponce S, Jones-Smith JC. Pathways of the association between maternal employment and weight status among women and children: qualitative findings from Guatemala. Maternal & Child Nutrition. 2018;14: e12455. doi: 10.1111/mcn.12455. |
|  | Ojeda VD, Magana C, Burgos JL, Vargas-Ojeda AC. Deported men’s and father’s perspective: the impacts of family separation on children and families in the U.S. Frontiers in Psychiatry. 2020;11: 148. doi: 10.3389/fpsyt.2020.00148. |
|  | Panico L. Family structure and child health. PhD Thesis, University College London. 2012. Available from: <https://pdfs.semanticscholar.org/a4f7/553d1fbf4257989f5f372e7593c7c94a1216.pdf>. |
|  | Panico L, Bartley M, Kelly YJ, McMunn A, Sacker A. Family structure trajectories and early child health in the UK: pathways to health. Social Science & Medicine. 2019;232: 220-229. doi: 10.1016/j.socscimed.2019.05.006. |
|  | Park J. Predictors of health-promoting behaviors in children from low-income families: an ecological approach. Nursing & Health Sciences. 2018;20: 60-68. |
|  | Parveen S, Nasreen S, Allen JV, Kamm KB, Khan S, Akter S, et al.. Barriers to an motivators of handwashing behavior among mothers of neonates in rural Bangladesh. BMC Public Health. 2018:18: 483. doi: 10.1186/s12889-018-5365-1. |
|  | Pierce JS, Kozikowski C, Lee JM, Wysocki T. Type 1 diabetes in very young children: a model of parent and child influences on management and outcomes. Pediatric Diabetes. 2017;18: 17-25. doi: 10.1111/pedi.12351. |
|  | Repetti RL, Taylor SE, Seeman TE. Risky families: family social environments and the mental and physical health of offspring. Psychological Bulletin. 2001;128(2): 330. |
|  | Riesch SK, Anderson LS, Krueger HA. Parent–child communication processes: preventing children's health‐risk behavior. Journal for Specialists in Pediatric Nursing. 2006;11(1): 41-56. |
|  | Robila M. Parental migration and children’s outcomes in Romania. Journal of Child and Family Studies. 2011;20(3): 326-333. |
|  | Roosa MW, Dumka L, Tein JY. Family characteristics as mediators of the influence of problem drinking and multiple risk status on child mental health. American Journal of Community Psychology. 1996;24(5): 607-624. |
|  | Rooth H. All parents in focus: governing parents and children in universal parenting training. PhD Dissertation, Mälardalen University. 2018. Available from: <http://www.diva-portal.org/smash/get/diva2:1256950/FULLTEXT02.pdf>. |
|  | Sasser J, Duprey EB, Oshri A. A longitudinal investigation of protective factors for bereaved maltreated youth. Child Abuse & Neglect. 2019;96: 104135. doi: 10.1016/j.chiabu.2019.104135. |
|  | Scheinfeld E, Shim M. Understanding eating behaviors through parental communication and the integrative model of behavioral prediction. American Journal of Health Behavior. 2017;41(3): 228-239. doi: 10.5993/ajhb.41.3.2. |
|  | Schor EL, Menaghan EG. (1995). Family pathways to child health. In: Amick III BC, Levine S, Tarlov, AR, Walsh DC, editors. Society and Health. New York: Oxford University Press; 1995. pp.18-45. |
|  | Shapiro GK, Tatar O, Amsel R, Prue G, Zimet GD, Knauper B, et al. Using an integrated conceptual framework to investigate parents’ HPV vaccine decision for their daughters and sons. Preventive Medicine. 2018;116: 203-210. doi: 10.1016/j.ypmed.2018.09.017. |
|  | Smith EP, Gorman-Smith D, Quinn WH, Rabiner DL, Tolan PH, Winn DM, et al. Community-based multiple family groups to prevent and reduce violent and aggressive behavior: the GREAT Families Program. American Journal of Preventive Medicine. 2004;26(1): 39-47. |
|  | Soubhi H, Potvin L. Homes and families as health promotion settings. In: Poland BD, Green LW, Rootman I, editors. Settings for health promotion: Linking theory and practice. Thousand Oaks, California: SAGE Publications Inc.; 2000. pp. 44-86. |
|  | Swindle TM, Jarrett D, McKelvey LM, Whiteside-Mansell L, Connors Edge NA, Kraleti S. Test of a conceptual model to explain television exposure of Head Start Children. Clinical Pediatrics. 2018:57(8): 970-980. doi: 10.1177/0009922817738340. |
|  | Taylor RD, Roberts D. Kinship Support and maternal and adolescent well-being in economically disadvantaged African-American families. Child Development. 1995;66(6): 1585-1597. |
|  | Tollefson MM, Finnie DM, Schoch JJ, Eton DT. Impact of childhood psoriasis on parents of affected children. Journal of American Academy of Dermatology. 2017;76: 286-289. doi: 10.1016/j.jaad.2016.09.014. |
|  | Travis S, Bisogni C, Ranzenhofer L. A conceptual model of how US families with athletic adolescent daughters manage food and eating. Appetite. 2010;54(1): 108-117. |
|  | Travlos V, Downs J, Wilson A, Hince D, Patman S. Mental wellbeing in non-ambulant youth with neuromuscular disorders: what makes the difference? Neuromuscular Disorders. 2019;29: 48-58. doi: 10.1016/j.nmd.2018.08.013. |
|  | Trost SG, Sallis JF, Pate RR, Freedson PS, Taylor WC, Dowda M. Evaluating a model of parental influence on youth physical activity. American Journal of Preventive Medicine. 2003;25(4): 277-282. |
|  | Vaughn AE, Hales DP, Neshteruk CD, Ward DS. HomeSTEAD’s physical activity and screen media practices and beliefs survey: instrument development and integrated conceptual model. PLoS ONE. 2019;14(12): e0226984. doi: 10.1371/journal.pone.0226984. |
|  | Wade C, Llewellyn G, Matthews J. Parent mental health as a mediator of contextual effects on parents with intellectual disabilities and their children. Clinical Psychologist. 2015;19(1): 28-38. doi: 10.1111/cp.12055. |
|  | Wise S. Family structure, child outcomes and environmental mediators: an overview of the Development in Diverse Families study. Australian Institute of Family Studies. 2004;30: 1-42. |
|  | Wood BL, Miller, BD. Families, health, and illness: the search for pathways and mechanisms of effect. In: Pinsof WM, Lebow JL, editors. Family Psychology: The Art of the Science. New York: Oxford University Press; 2005. pp. 493-520. |
|  | Zeitlin MF, Megawangi R, Kramer EM, Colletta ND, Babatunde ED, Garman D. Strengthening the family: Implications for international development. Tokyo: United Nations University Press; 1995. |
|  | Zhang Y, Kar YL, Edward CML, May CMW. Structural equation model for parental influences on children’s oral health practice and status. BMC Oral Health. 2020;20: 56. doi: 10.1186/s12903-020-1048-2. |
|  | Zheng K, Bruzzese J-M, Smaldone A. Illness acceptance in adolescents: a concept analysis. Nursing Forum. 2019;54: 545-552. doi: 10.1111/nuf.12368. |
