# Supplementary material for: Family as a health promotion setting: A scoping review of conceptual models of the health-promoting family
Source: PLoS One. 2021 Apr 12;16(4):e0249707. doi: 10.1371/journal.pone.0249707 (PMC8041208; doi:10.1371/journal.pone.0249707)
Supplement: S2 Table — (DOCX) [file pone.0249707.s002.docx]

**S2 Table. Environmental and/or ecological factors detailed in models.**

**List of environmental and/or ecological factors in according to model.**

| # | Model name | Ecological and environment factors as described in model | Source (See references for full citation) |
| --- | --- | --- | --- |
| 1 | Figure 8.3. Supportive factors and mothers’ agency in the school environment. | Education; self-efficacy; community programs; mother’s agency | Aldossari, 2016 [17]. |
| 2 | Figure 2. Conceptual framework on malnutrition. | Height/weight for age, feeding practices, child’s initial health status, disease, household characteristics, maternal characteristics, structural factors | Annim, Awusabo-Asare, & Amo-Adjei, 2015 [18]. |
| 3 | Figure 1. Conceptual model. | Environmental, personal stressors and characteristics, social supports, proximal outcomes (parental wellbeing, quality of parenting), self-esteem, strengths, health, competence and empowerment. | Armstrong, Birnie-Lefcovitch, & Ungar, 2005 [19]. |
| 4 | Figure 1. Model linking income, material hardship, and parenting to child health status. Figure 2. SEM. | Model linking Income, Material Hardship, and Parenting to Child Health Status. | Ashiabi & O'Neal, 2007 [20]. |
| 5 | Figure 1. Conceptual model for SOL youth: Understanding risk and protective factors for Latino childhood obesity. | Community, organizational, interpersonal, individual-Children and parents are reporting on individual, interpersonal, and perceived organizational and community influences on children’s risk for obesity consistent with Socio-Ecological Framework. | Ayala et al., 2014 [21]. |
| 6 | Figure 1. Expected associations among family communication orientations, health-specific conversation factors, and health outcomes. Figure 2 Multilevel structural equation model (SEM). | Social ecological, family conversation, family conformity, talk freq., per confirm=health attitudes=health behaviour | Baiocchi-Wagner & Talley, 2013 [22]. |
| 7 | Figure 2. Potential mediating role of father in relationships linking health determinants to child health;  Figure 3. Research evidence of direct effects of father’s involvement on child development;  Figure 4. Research evidence of direct effects of father’s absence on child development; Figure 6. Reciprocally causal links among health determinants and outcomes of father’s involvement. | Ecological conceptualization of determinants of health- The conceptual framework outlined below identifies some of features of an ecological system of core dimensions and dynamics that could be explored in order to investigate both the direct and indirect ways that fathers can contribute to children’s health and development. Background conditions, determinants of healthy child development from community, from family unit, from father’s interaction with child | Ball, Moselle, & Pedersen, 2007 [23]. |
| 8 | Fig. 1. Final two-factor oblique confirmatory factor analysis model of family entropy with standardized parameter estimates. | Family home environment; organization; disorganization; household characteristics | Bates et al., 2019 [24]. |
| 9 | Figure 1. An integrated model of social environment and social context. | Community; neighbourhood; organizations/institutions; groups/networks; resources; supports; cultural; economic; social environment; technological; social context; neighbourhood; school zone | Batorowicz et al., 2016 [25]. |
| 10 | Figure 1. Theoretical cascade model linking provider delivery, participant responsiveness, and improvements in program outcomes. | Home practice; culture; parent gender | Berkel et al., 2018 [26]. |
| 11 | Figure 2-3. Model of community nutrition environment (Glanz, Sallis, Saelens, & Frank, 2005). | Food access; food availability; affordability; nutritional quality of food; nutrition information; food quality; nutrition promotion; nutrition policies; demographics,; diet | Bertrand, 2019 [27]. |
| 12 | Figure 2-4. Ecological framework depicting the multiple influences on what people eat (Story, Kaphingst, Robinson-O’Brien, & Glanz, 2008). | Home environment; neighbourhood and community; access to supermarkets; school policy; cognitions; skills and behaviours; lifestyle; gender; genes; demographics; family environment; peers; friends; societal and cultural norms and values; food policy; school food policy; government nutrition policy; health care system; access to transportation; nutrition communication; food and agriculture policies; food assistance programs | Bertrand, 2019 [27]. |
| 13 | Figure 1-1. Realms of family life: A focus of family health nursing practice. | Family coping, family realms, family interactive processes, family system health maintenance, family integrity processes, family developmental processes | Bomar, 2004 [28]. |
| 14 | Figure 1. Adaptation phase of the resiliency model of family stress, adjustment, and adaptation. | Interpersonal relationships, development wellbeing and spirituality, community relationships and nature, structure, family resources, | Brown, Fouche, & Coetzee, 2010 [29]. |
| 15 | Figure 2. The ENERGY- project specific ENRG (Environmental Research for weight Gain prevention) framework. | Cognitive mediators, moderators, family and school environment=EBRB | Brug et al., 2010 [30]. |
| 16 | Fig.1 Conceptual model of social determinants of health and racial/ethnic disparities in T2DM. (Adapted with permission from: Walker RJ et al. BMC Endocr Disord. 2014;14:82; with permission from BioMed Central) | Social determinants; SES; low level of parental education; high stress in youth; race; ethnicity; family coping; family health knowledge; access to care; quality of care | Butler, 2017 [31]. |
| 17 | Figure 1. Conceptual Model 1 with caregiver-adolescent discrepancies.  Figure 2. Conceptual Model 2 with adolescent and caregiver acculturation main effects. | Family functioning; acculturation; government practices; self-identity; ethnicity; health risk taking behaviors; family dimension; | Cano et al., 2016 [32]. |
| 18 | Figure 3.5. Proposed integrated conceptual model for the understanding pathways that influence child development and the impact of child health on the family. | Social ecological-maternal characteristics, family factors and resources, community health (physical environment and social environment) | Cheng, 2013 [33]. |
| 19 | Figure 1. Bruhn and Parcel model of health promotion (1982). | Family influence; family interaction; constraints and opportunities; age; intelligence; coping behaviors; cognitive style; health beliefs; self-identity; self-efficacy; disease; cognitive development | Chiu, 2005 [34]. |
| 20 | Figure.1. Conceptual framework based on the theory of planned behavior: Factors that influence children’s beverage consumption behaviors. | Race; ethnicity; SES; parent education; family structure; parent health behaviors; perceived behavioral control; self-efficacy; gender; age; health beliefs; family interactions; family beverage practices; child temperament; household rules; neighbourhood; access to healthy food; media/advertising; policies | Choy & Isong, 2018  [35]. |
| 21 | Figure 1. Model of the health-promoting family. | Consumer society, local community, friends and peers, school, day care, health services, media; family | Christensen, 2004 [10]. |
| 22 | Figure 1. Summary of themes and subthemes identified in qualitative interviews. | Health beliefs; family health practices; parent’s physical activity; parents’ commitment to child PA; access to physical activity programming; parents’ teaching proficiency of PA | Columna et al., 2019  [36]. |
| 23 | Figure 2.1. Child-parent reciprocal influences model. | Self-efficacy; health beliefs; family structure; family flexibility; family cohesion; SES; social support; parent biological and personal characteristics; child biological and personal characteristics | Coviak, 1998 [37]. |
| 24 | Figure 5.1. The model of concept of well-being in older Taiwanese. | Family support; self-dignity; social support; family variables (obligations, support, well-being); social environment | Dai, 1995 [38]. |
| 25 | Figure 5.1. A graphical depiction of the impact of family processes on children's emotional insecurity in the family and their trajectories of adjustment within the reformulated emotional security theory. | Parenting practices; parent-child insecurity; family processes; child attributes; interparental conflict; interparental insecurity | Davies, Sturge-Apple, & Martin, 2013 [39]. |
| 26 | Figure 2. The revised family ecological model; bolded text and boxes indicate new components and constructs that were not part of the original model. | Family ecological model; family history and structure; child-specific characteristics; organizational factors; community factors; media and policy factors; family and emotional context; family knowledge and social norms; SES; food insecurity; parenting practices and behaviours related to healthy lifestyle; health beliefs; diet; physical activity | Davison, Jurkowski, & Lawson, 2013 [40]. |
| 27 | Figure 1. Structural model linking mothers' gendered roles and ideologies to adolescent depression. Figure 2 (SEM modelling). | Maternal employment; gender; poverty; family income; age; sex; behavior problems; ethnicity; race; female-headed household | De Coster & Zito, 2013 [41]. |
| 28 | Fig. 1. Social-ecological model applied to the Kanyakla Nutrition Program (Gregson et al., 2001; Stokols, 1992). | Social-ecological model; individual factors; personal resources; health beliefs; attitude; family support; friends; neighbourhood; culture; values; community factors; access to community health workers, health clinics, churches; regional fisheries, local markets; policy; infrastructure; laws; natural resources | DeLorme et al., 2018  [42]. |
| 29 | Fig 2. Conceptual model of the Kanyakla nutrition program. | Community network; nutrition knowledge/education; nutrition knowledge; community support; family support; maternal nutrition behaviour; child nutrition behaviour; household food security | DeLorme et al., 2018  [42]. |
| 30 | Figure 1-1. Social construction of family health. | Ecological framework; household; social, historical, political aspects; family health routines; health behaviours; family processes; Race; culture; age; gender; education; SES; family health routine; member relationships; eco cultural domains | Denham, 2003 [43]. |
| 31 | Figure 9-1. Social construction of family health definitions and practices. | Family function and context; ecocultural domains; family health practices; family routines; environmental niches; family relationships | Denham, 2003 [43]. |
| 32 | Figure 12-1. Factors affecting the modification of the family health constructs. | Parental beliefs; family traditions; health experiences; illness/disease; family life; family development; ecological context; relocation; job loss; substance abuse; accidents; extended family support; social relationships; knowledge exposure; education | Denham, 2003 [43]. |
| 33 | Figure. Theoretical model adopted in the study. | Gender; SES; home environment; parent health beliefs; child health beliefs | dePaula et al., 2015 [44]. |
| 34 | Figure 11.1. Family formation in low-income populations. | Mother, father personal characteristics; age; race; ethnicity; parent relationships; fertility; domestic violence; health beliefs and attitudes; education; employment; health; parenting behaviour; kin support; family function; parenting behaviour; cognitive stimulation; family structure | Dion et al., 2003 [45]. |
| 35 | Fig. 1. The relationship between parent-child, stimulation and dental caries: a life course approach. | SES at birth; mother-child interaction; access to health care; maternal employment; maternal mental health; parental warmth; parent-child interaction; home environment | dos Santos Costa et al., 2019 [46]. |
| 36 | Figure 1. Conceptual model of influences on adherence to paediatric asthma treatment. | Race; income; parental education; family routine; family communication; child age; child knowledge of illness; child health/illness; parent knowledge; parent health/illness; access to health care; quality of medical care; health communication | Drotar, & Bonner, 2009 [47]. |
| 37 | Figure 1. Final structural equation model relating latent constructs of neglect to children's functioning. | Parental support; family constructs; family conflict; maternal support and affection; family conflict; child anxiety and depression; social problems; parent attachment; father present; home-cooked meals | Dubowitz et al., 2005 [48]. |
| 38 | Figure 1. Schematic illustration of the theoretical model. | Maternal education; family organization; parental self-efficacy; parental locus of control; disease | Duijster et al., 2014 [49]. |
| 39 | Figure 1. Theoretical model of chaos and child health. | Household chaos; work-family-child care chaos; environmental exposures; stress; lack of emotional support; lack of prevention; treatment of illness; inadequate supervision; family resources for housing; more need to work; less parental time; more family stress; less consistent child care | Dush, Schmeer, & Taylor, 2013 [50]. |
| 40 | Figure 1. Conceptual model; Figure 2. Model for female and male younger siblings (SEM); Figure 3. Model for female younger siblings (SEM)]. | Sibling alcohol dependency; sibling health risk behaviours; maternal involvement; family composition; age; gender; race; ethnicity; sibling age, gender, race, ethnicity; receipt of aid | East & Khoo, 2005 [51]. |
| 41 | Figure 2-1. Conceptual framework for reviewing ‘obesogenic landscapes’ in urban children’s geographies. | Ecological environment and environmental exposure; neighbourhood; urban or rural; healthy physical environment; transportation; city; safety; urban design | Ergler, 2012 [52]. |
| 42 | Fig. 1. Thematic analysis. | Health literacy; access to health information; health communication; access to reliably information | Fairbrother et al., 2016 [53]. |
| 43 | Figure 2. Modified model predicting family adaptation. | Family resources; social support; education; economic resources; family adaptation; coping strategies | Farhood, 1999 [54]. |
| 44 | Figure 1. Child, family, and community influences on oral health outcomes of children. | Family composition; family-level influences; SES; social support; physical safety; health status of parents; family function; culture; health beliefs; coping skills; biological and genetic endowment; physical and demographic attributes of child; health behaviours; dental insurance | Fisher-Owens et al., 2007 [55]. |
| 45 | Figure 1.2. Pathways of family processes;  Figure 1.3. Life process of the family system. | Family congruence; culture; health values and beliefs; safety; emotional support; culture and tradition; family communication; social skills; family values; family system maintenance; family system change; personal development | Friedemann, 1995 [56]. |
| 46 | Figure 1. The PEN-3 cultural model. | Culture identity; relationships; family; support networks; knowledge; health beliefs; community; environment | Galbraith-Gyan et al., 2019 [57]. |
| 47 | Figure 1. Familial approach to the treatment of childhood obesity: conceptual model. | Parental nutrition/health skills; parenting skills; healthy environment in family home; child weight; parent nutrition knowledge; resources for health foods; companionship at meal times; self-efficacy in parent food habits; parent exercise patterns; parent role; effective communication; problem-solving skills; regular meal times; leisure time activities; access to outdoors | Golan & Weizman, 2001 [58]. |
| 48 | Figure 1. Adapted from the transactional stress and coping (TSC) model of adjustment to chronic illness (Thompson et al. 1994) for siblings. | Demographic parameters; sibling gender, age, grade level; SES; Family parameters: extended family size; Family adaptational processes: family coping, family functioning; sibling coping, self-efficacy, perceived social support | Gold et al., 2008 [59]. |
| 49 | Fig. 1. FRESH theoretical model. | Education; physical activity opportunities; health communication | Guagliano et al., 2019a [60]. |
| 50 | Figure 3. FRESH theoretical model. FRESH, Families Reporting Every Step to Health. | Family support; family PA norms; physical activity awareness/education; family physical activity; health behaviours | Guagliano et al., 2019b [61]. |
| 51 | Fig.1 A conceptual model of influence of family dynamics and sleep health behaviors on hypertension risk. | Family relationships; family dynamics; family relationship quality; health behaviours; BP; BMI; HRV | Gunn & Eberhardt, 2019 [62]. |
| 52 | Figure 1. | Tragic life event; bereavement processes; emotional stability; coping; emotional security; interparental conflict | Hardt et al., 2019 [63]. |
| 53 | Figure 1. Conceptual model for predictors of children's development. | Child characteristics; diagnosis, gender, health; parental education, marital status; Child self-regulatory process; Family climate; mother-child interaction, family relations | Hauser-Cram et al., 2001 [64]. |
| 54 | Figure 2. Conceptual model for predictors of parent well-being. | parental education; marital status; parent assets; problem-focused coping; social support; child characteristics; type of disability; gender; health; family climate; mother-child interactions; family relations; child skills; developmental skills (cognitive and adaptive skills); self-regulatory processes (mastery motivation, behavior regulation) | Hauser-Cram et al., 2001 [64]. |
| 55 | Figure 1. Conceptual Model of the Influence of Macro- and Family-Level Sociocultural Contextual Factors in Youth and Pubertal Timing on Women’s Lifetime Educational Achievement. | Biological life event/time of menarche; geographic region; race; ethnicity; year of birth; father absence, mother; educational attainment; mother’s age at first birth; sociocultural factors; health behaviours | Hendrick et al., 2016 [65]. |
| 56 | Figure 1. Obesity resistance model: a summary of the interactions of family environmental factors influencing children's weight status and behaviors. | Parent exercise, BMI, knowledge, diet quality; family environment; quality of food; physical activity; parenting style; child feeding practices | Hendrie, Coveney & Cox, 2012 [66]. |
| 57 | Figure 1. Relationship-based feeding framework. | Child caregiver relationship; community support and resources; social influences; mealtimes; child-caregiver relationship; education; biopsychosocial factors | Henton, 2018 [67]. |
| 58 | Figure 1. Model illustrating the mediation paths for the combined sample (top panel), ASD+parent-reported below average IQ (middle panel), and ASD+parent-reported average or above IQ (bottom panel) between ASD severity, parental romantic expectations for their child, and number of sex- related topics covered by parent. | Parent IQ; child IQ; disease; gender; parents’ perceived child efficacy; parent-child communication | Holmes et al., 2016 [68]. |
|  | Figure 1. Conceptual framework of life events and cultural processes that shape maternal capabilities and influence child nutrition and hygiene care behaviors. | Social support; physical health; family relations; maternal intrinsic capabilities; family planning/decision-making; partner support; early marriage; early pregnancy | Ickes et al., 2017 [69]. |
| 59 | Fig. 1. Social ecological model applied to child health (Kazak, 2006). Fig. 2. Coding tree, based on the social ecological model applied to child health by Kazak (2006). | Schools; technology; social class; social network; peers; access to hospitals; cultures; religion; law; neighbourhoods; parents; siblings; family cohesion; illness | Janin et al., 2018 [70]. |
| 60 | Figure 1. Empirical model that summarizes the study’s findings, based on the adolescents’ voices and the researchers’ interpretation of the empirical data through self-determination theory (SDT). | Support from family and friends; physical skills; friends; autonomy; supportive school environment; physical activity opportunities | Jonsson et al., 2017 [71]. |
| 61 | Figure 1. “Influence of child, family, and community on oral health outcomes of children” (Fisher-Owen et al., 2007). | Community level influences; social environment; dental care system; physical safety; physical environment; community health environment; social capital; culture; family level influences; SES; social support; physical safety; health status of parents; family function; health behaviors, practices, coping skills; biologic and genetic endowment; physical and demographic attributes; health insurance | Kalil, 2017 [72]. |
| 62 | Figure 1. Proposed mediation model; Figure. 2. Direct model without mediation; Figure. 3. Full mediation model. | Parent health-related feeding goals; parent health; natural foods; parent negative feeding practices (feeding child for emotional regulation, child control, or food reward) | Kiefner-Burmeister et al., 2014 [73]. |
| 63 | Figure 1. A unifying conceptual model for early childhood caries (ECC) showing the connections between social, environmental, maternal, and child factors. | Maternal traits; low education levels, psychological distress (anxiety & depression); health beliefs (with external locus of control); family stress; dysfunctional parenting; difficult child temperament | Kim Seow, 2012 [74]. |
| 64 | Box 9-1. Characteristics of Healthy Family. | Family commitment; traditions; trust; family rituals; leisure time; positive communication; family interaction; family values; adaptability; | Kim-Godwin & Bomar, 2010 [75]. |
| 65 | Figure 1. A model of factors affecting the participation of children with disabilities. | Absence of financial and time impact on family; supportive family demographics; supportive home environment; family preference for recreation; child: self-perceived competence, physical, cognitive, & communicative function, emotional, behavioural, & social function, activity preference; disability; SES; child recreational activities; physical activities | King et al., 2003 [76]. |
| 66 | Figure 1. Family systems theory framework related to youth health behaviors. | Family systems variables; family competence to manage daily tasks in context of supportive environment; family satisfaction; family warmth; caring and nurturance between family members; family cohesion; emotional bonding between family members;  positive (authoritative) parenting styles; shared decision making; setting appropriate boundaries; effective conflict resolution; appropriate monitoring for developmental stage; warm and supportive parental behaviours; diet; physical activity | Kitzman-Ulrich et al., 2010 [77]. |
| 67 | Figure 2. Model for family psychosocial well-being in a South African context. | Family functioning; family strengths; family (parents, guardians, children, extended family); internal and external influences: [Negative] financial difficulties, conflict, absent father/ husband, not enough time together, isolation/ few friends, poor communication, negativity, unsupportive family; internal and external influences: [Positive] togetherness, effective communication, spirituality, mutual support, respect, acceptance/ understanding, healthy/ safe environment, education, problem solving, discipline, loving each other, responsibilities/ chores, parental guidance, honesty/ trust; friendships; education; community; safe environment | Koen, van Eeden, & Rothmann, 2013 [78]. |
| 68 | Figure 1. Conceptual framework explaining the relationship between family structure, number of siblings and child well-being. | Family structure; number of siblings; family resources; presence of care giver/ decision-makers other than parents; distribution of financial resources, time, energy | Kumar & Ram, 2013 [79]. |
| 69 | Figure 1. Theory of change of M-PACT+ | Family relations; communication; sources of support; addiction; parental responsibility; self-esteem; school staff; parental physical activity; education | Laing et al., 2019 [80]. |
| 70 | Figure 2.3. Conceptual framework for the development of nurse-led health promotion visiting programme and family health. | Resource availability; access to health care practitioners | Lam, 2016 [81]. |
| 71 | Figure 1. Conceptual model of how parents influence their child's dietary behavior. | Food parenting practices; parental dietary behaviour; parenting consent; child's home food environment (food availability, modeling); child's characteristics; parenting style; differential parenting practices; child’s dietary behaviour | Larsen et al., 2015 [82]. |
| 72 | Fig. 1. Conceptual framework of automatic and underlying techniques that may bridge the intention-behavior gap in food parenting. | Food parenting intentions; food parenting behaviours; parental food habits | Larsen et al., 2018  [83]. |
| 73 | Figure 1. Integrative model for understanding acculturation and Latino adolescent mental health. | Family factors; parenting behaviour; family conflict; individual factors: acculturative stress, coping; parental/ adolescent acculturation: cultural practices, values, identification | Lawton & Gerdes, 2014 [84]. |
| 74 | Figure 2 Influences on PA and sedentary behaviors of preschool-age children organized within the social ecological model. Adapted from McLeroy et al. (1988) | Physical activity policy influences; physical activity policy; government regulations; organization influences; child care environment; environmental influences; weather and season; availability and access to park and recreational facilities; community and neighborhood safety; interpersonal influences; parental beliefs and attitudes; SES; acculturation; ethnicity; age; gender | Lindsay et al., 2017 [85]. |
| 75 | Figure 1. The theoretical frame that will be modeled by using SEM. | Child characteristics (age, gender, number of siblings); family financial strain; parental education; mothers well-being; marital relationship; parenting behaviors | Liu, 2003 [86]. |
| 76 | Figure 1. Model for Spanish Adolescents.  Figure 2. Model for Immigrant Adolescents. | Self-esteem; family support; psychological adjustment; ethnicity | Lopez-Rodriguez et al., 2018 [87]. |
| 77 | Figure 1. Theoretical model linking co-parenting and parent and child anxiety. | Extrafamilial strain/support, couple relationship; coparenting: support/ undermining; agreement on childrearing; task division; family interaction; child emotional security; parenting: overcontrol, negativity, lack of challenging behaviour | Majdandžić, et al., 2012 [88]. |
| 78 | Figure 1. Conceptual model of the association between fathers’ involvement and individual psychosocial health outcomes mediated by family flexibility and moderated by marital quality. | Marital quality; father involvement; military context; family flexibility | Mallette et al., 2020 [89]. |
| 79 | Figure 1. Coding schema derived from original model of adolescent asthma self-management | Knowledge; cognitive characteristics; family environment; social environment; physical environment; support; communication; access to healthcare; caregiver | Mammen et al., 2018 [90]. |
| 80 | Figure 4. Revised model of self-management, with delineation of subcomponents specific to asthma and adolescents. | Knowledge/education; access to healthcare; disease | Mammen et al., 2018 [90]. |
| 81 | Figure 1. The six metathemes of family preparedness, based upon thematic analysis of interviews with families and clinicians. | Health communication; education; care coordination; access to care; quality of care; family background; support systems; coping skills | Markwalter et al., 2019 [91]. |
| 82 | Figure 1. Partial indirect effects model. | Maternal/paternal support; adolescent ADHD symptoms; parent involvement; parent autonomy | Meinzer et al., 2015 [92]. |
| 83 | Fig. 1. Conceptual model of hypothesized benefits of a bedtime routine. | Family routine; consistent routine; hygiene; communication; nutrition; health literacy; parent-child attachment; family stress; health beliefs | Mindell & Williamson, 2018 [93]. |
| 84 | Figure 1. Theoretical model of children’s developing health lifestyles. | Background; resources; family health habits; school; age; maternal age; SES; social resources | Molborn & Lawrence, 2018 [94]. |
| 85 | Figure 1. Phenomena and categories-Campinas, 2016/2017. | Extended family support; family support; illness; hospital; quality of care; health information; resources | Moraes & Mendes-Castillo, 2018 [95]. |
| 86 | Figure 1. Levels of interacting family environmental subsystems (LIFES). | Family functioning; family physical activity climate; personality; intrinsic motivation; parental beliefs about child physical activity; self-efficacy; parent participation in physical activity; modelling by parent; cognitive factors; gender; parenting practices; parent-child interactions; family priorities | Niermann et al., 2018 [96]. |
| 87 | Figure A7.3. Theme material conditions with categories and concepts. | Foodscape; work patterns; conditions of pay; time constraint for caregiving; affordability; family composition; family dynamics; working parents; stay at home mom; food poverty; food quality | Noonan-Gunning, 2018 [97]. |
| 88 | Figure 1. Pathways by which maternal employment may play a role in maternal and child weight status. | Increased income; employment; childcare; food purchasing; household well-being; time spent on preparing and purchasing meals; maternal employment; household tasks; time for supervision | Oddo et al., 2018 [98]. |
| 89 | FIGURE 1 \| A framework for researching the outcomes of family separation due to paternal deportation. | Deportation policy; income; aren’t-child relationship; parent-parent relationship; parental social ties; community; public institutions; immigration/citizen status; age; gender; education; employment; social networks; single-parent household; SES | Ojeda et al., 2020 [99]. |
| 90 | Figure 3.1. Initial conceptual model. | SES; family structure; family activities; social networks; physical environment; emotional environment; economic environment; child behaviour | Panico, 2012 [100]. |
| 91 | Fig. 1. Conceptual model. | SES; physical environment; emotional environment; health behaviours; economic environment; family structure | Panico et al., 2019 [101]. |
| 92 | Figure 1. Conceptual model for predicting the health-promoting behaviors of children from low-income families. | Access to community child care centre; quality of community child care centre; perceived physical environment of child care centre; teachers workloads; length of child care centre establishment; family structure; caregivers education level, weight status, parenting behaviour; peer relationships; child sex, weight status, self-efficacy, self-regulation | Park, 2018 [102]. |
| 93 | Fig. 1. Conceptual model to explain motivations of maternal handwashing behavior in the neonatal period. | Self-efficacy; health behaviours; perceived and normative health beliefs; mothers; fathers; secondary caregivers; hygiene behaviours; physical environment; social dimensions | Parveen et al., 2018 [103]. |
| 94 | Fig. 1. A conceptual model linking individual parent characteristics, parental coping, and individual child characteristics to the management and outcomes of type 1 diabetes (T1D) in very young children (YC-T1D). | SES; social support; parenting style; family history; parent affective, behavioral and cognitive coping; child temperament; child age, development, illness, disease; health care communication | Pierce et al., 2017 [104]. |
| 95 | Figure 1. Risky families model. | Family social context; genetic factors; risky family social environment (conflict and aggression, cold, unsupportive, and neglectful home); child emotion processing, social competence; adolescent risky health behaviors (substance abuse, risky sexual behaviour) | Repetti, Taylor, & Seeman, 2002 [105]. |
| 96 | Figure 1. Conceptual model for reducing health-risk behaviors in middle childhood. | Family nonmodifiable demographics; partnered status/harmony; education; race; age; income and occupation; family parenting practices (modifiable): regulation/structure, involvement/monitoring, school connectedness, communication of expectations, models of health, behavior, and practices); individual (child) factors: nonmodifiable (pubertal development, brain maturity, hormones); modifiable (academic achievement, risk status, self-esteem, psychological autonomy, church & school attendance, number of friends); family communication processes: parent child communication (communication perceptions, family satisfaction, family caring, effective problem solving skills); health risk behaviour participation; unintentional injury; risk behaviours | Riesch, Anderson, & Krueger, 2006 [106]. |
| 97 | Figure 1. Conceptual model SEM; Figure 2. Results for the model with satisfaction with migration; Figure. 3 Results for the model with desired migration. | Migration; SES; family conflict; parenting behaviours; educational achievement; parenting quality; parent conflict; child academic achievement | Robila, 2011 [107]. |
| 98 | Figure 1. Theoretical stress process model with family cohesion and family reframing coping as mediators of the influence of family drinking problems and multiple family risks on child mental health with hypothesized direction of relationship arrows; Figure 2. SEM. | Family cohesion; family reframing coping; family drinking problems; family multiple risk; child gender; negative life events | Roosa, Dumka, & Tein, 1996 [108]. |
| 99 | Figure 2. An illustration of how ontological and epistemological choices lead to different routes in universal parenting training. | Risk-based preventive parenting policies; parent and child knowledge; policy; training; children’s welfare rights; parenting skills; parenting training | Rooth, 2018 [109]. |
| 100 | Fig. 2. Path analysis model of the moderating effect of future orientation (family) on the association between bereavement and externalizing problems. Fig. 3 and 4. Path analysis model of the moderating effect of parent-child relationship (Fig. 3) and parental monitoring (Fig. 4) on the association between bereavement and externalizing problems. | History of child maltreatment; individual protective/risk factors; family protective/risk factors; community protective/risk factors; SES; death | Sasser et al., 2019 [110]. |
| 101 | Figure 1. Hypothesized research model  Figure 2. Model with standardized Beta values.  Figure 3. Model with statistically significant pathways. | Parent-child communication frequency, quality, content; eating behaviours; health attitude/beliefs; perceived behavioural control; parent health beliefs; parental norms | Scheinfeld & Shim, 2017 [111]. |
| 102 | Figure 2-1. Social context of child health. | Child physical, social, emotional, educational, health behaviours; family functioning; family environment; family functions; social network; extended family, friends/ neighbors/ colleagues; community norms, values, resources; social policy; family support; health care; education; family characteristics; biological factors; health behaviours; SES; family structure; marital status; family composition; family relations; child biological characteristics; family life cycle; child development; child’s community support; race; language; ethnicity | Schor & Menaghan, 1995 [112]. |
| 103 | Fig. 1. An integrated conceptual framework of HPV vaccination. | Child’s gender, age, language, ethnicity, vaccine knowledge, health care provider; health beliefs; parent gender, age, language, marital status, level of education, employment status, citizenship, ethnicity, religion, household income; school; size of city | Shapiro et al., 2018 [113]. |
| 104 | Figure 1. Aspects of family life. | Family cohesion; family beliefs; family relationships; neighbourhood support; school; marital status; family organization; parent involvement; social contexts | Smith et al., 2004 [114]. |
| 105 | Figure 2.1. Social context of family health. | Family health; family health promotion; family characteristics; family environment; family values and norms; social context; extended family support; family policy; housing; employment | Soubhi & Potvin, 2000 [115]. |
| 106 | Figure 1. Conceptual model of child television exposure.  Figure 2. Model estimates. | Neighbourhood quality; social support; ethnicity; education; employment status; marital status; urban; gender; sex | Swindle et al., 2018 [116]. |
| 107 | Figure 1. Conceptual model of the mediation of the association of kinship support and adolescent well-being.  [Figures 2, 3 and 4 are SEM testing of model components.] | Kin social support; maternal self-esteem, psychological distress; maternal acceptance; firm control; autonomy granting; adolescent functioning | Taylor & Roberts, 1995 [117]. |
| 108 | Fig 1. Conceptual framework of the effects of childhood psoriasis on parents of affected children. | Family function; social function; emotional well-being; parental concern about child; personal care; health care; money and finances; marital relationship; health awareness; burden of care; parent career; time; accommodation | Tollefson et al., 2017 [118]. |
| 109 | Figure 1. A conceptual model for family food systems in households with adolescent female athletes. | Family characteristics; ethnicity; social support; family structure; income; employment; family member needs; values; evaluation of food choice;  mother and adolescent daughter traits; family meal time patterns | Travis, Bisogni, & Ranzenhofer, L, 2010 [119]. |
| 110 | Fig 1. Questionnaire variables within the International Classification of Functioning, Disability and Health (ICF) framework (ACQoL – Adult Carer Quality of Life; HBSC – Health Behavior of School-age Children Study; MPSS – Measure of Perceived Social Support; MQ – Measurement Question; MSK – musculoskeletal; NIV – non-invasive ventilation; NMD – Neuromuscular Disorder; PEG – Percutaneous endoscopic gastrostomy, QYPP – Questionnaire of Young People’s Participation; WEMWBS – Warwick Edinburgh Mental Wellbeing Scale.) | Physical health; age; gender; academic achievement; condition specific knowledge; household composition; number of siblings; parental education; parental employment; external agency care assistance; friends and family support; parent adult carer; home life; relationships; education; work; autonomy; healthcare self-management; healthcare behaviours; health condition | Travlos et al., 2019 [120]. |
| 111 | Figure 1. Standardized parameter estimates for proposed theoretical model: “Model 1.” | Child age, gender; parental physical activity; parental enjoyment of physical activity; parental perceived importance of physical activity; parental instrumental support for physical activity; child self-efficacy perceptions | Trost et al., 2003 [121]. |
| 112 | Fig. 1. Conceptual model of parent physical activity and screen media practices and beliefs. | Physical activity practices; parent attributes; parent history; parent value for behavior; parent self-efficacy; parent perception of child PA attributes; permissive parent; structure in home; rules/structure; modelling; facilitation of PA for child | Vaughn et al., 2019 [122]. |
| 113 | Figure 1. Proposed model of influences on parent mental health, parenting practices and children of parents with intellectual disability;  Figure 2. Final model of influences on parenting and on children of parents with intellectual disability SEM. | SES; social supports; access to support; partner support; Parent mental health; parenting efficacy (at child care tasks); parenting warmth; parenting irritability | Wade, Llewellyn, & Matthews, 2015 [123]. |
| 114 | Figure 1. Mediation and moderation model of the relationship between family structure and child outcomes. | Family structure ; family transitions (number, timing); contextual factors; intrafamilial relations; parental functioning; child age, gender, temperament; parent interaction; social support; social context | Wise, 2004 [124]. |
| 115 | Figure 20.2. The biobehavioral family model. | Disease; space sharing; support; parent child attachment; family function; family relations; parent interactions; emotional climate of family; child attachment; child-parent dyads | Wood & Miller, 2005 [125]. |
| 116 | Figure 8.2. Detailed conceptual model based on Nigerian data. | Family resources; material resources; housing; budget; social resources; literacy; health communication; media; location; family management and beliefs; hygienic practices: clean, sanitary, water and sewer; feeding practices: food belief, food invest; affection & attention: warm, verbal, child care; academic stimulation: teaching, toys; child IQ; child development; developmental resources | Zeitlin et al., 1995 [126]. |
| 117 | Fig 1. Hypothesized path diagram of parent-child OHS model  Fig 2. Path diagram of family OHB model.  Fig 3. Path diagram of family OHS model.  Fig 4. Path diagram of mother-child OHS model. | SES; health knowledge and attitude; parent health behaviours; health status; parent health status; parent attitude towards children; children health behaviours; child health status | Zhang et al., 2020 [127]. |
| 118 | Figure 2. A conceptual model depicting the relationship between antecedents, attributes, and positive consequences of illness acceptance in adolescents | Illness acceptance; self-esteem; sense of identity; disease control; parental acceptance of child disease; peer support; family support; development readiness; disease education | Zheng et al., 2019 [128]. |
